# TRANSITIONS EXPERIENCED BY PEOPLE DIAGNOSED WITH DEMENTIA WHILE IN THE WORKFORCE

**DOI:** 10.1093/geroni/igac059.497

**Published:** 2022-12-20

**Authors:** Jennifer Boger, Sheida Marashi, Anna Mäki-Petäjä-Leinonen, Mervi Issakainen, Ann-Charlotte Nedlund, Charlotta Ryd, Louise Nygard, Arlene Astell

**Affiliations:** Waterloo University, Waterloo, Ontario, Canada; Waterloo University, Waterloo, Ontario, Canada; Insitute of Law & Welfare, Joensuu, Lappi, Finland; Institute of Law & Welfare, Joensuu, Lappi, Finland; Linkoping University, Linköping, Ostergotlands Lan, Sweden; Karolinska Institutet, Solna, Sodermanlands Lan, Sweden; Karolinska Institutet, Solna, Sodermanlands Lan, Sweden; University of Toronto, Toronto, Ontario, Canada

## Abstract

Dementia is a progressive, irreversible neurological disorder that causes changes in cognitive function and behaviour. While at least 5% of people who develop dementia every year are under the age of 65, dementia in the workplace is currently not well recognised or supported. The changes associated with dementia present multiple challenges for individuals who wish to continue with their employment. Many lose their positions before receiving a diagnosis, whilst others take sick or disability leave or early retirement. The process of understanding what is happening and coping with this new situation is highly individualistic and involves several transitions. The MCI@work project is an international initiative taking place in Canada, Finland, and Sweden where we examine these transitions through the personal narratives of individuals who have either currently or recently gone through the experience of developing dementia whilst in the workforce. From these data, we have developed a framework for understanding the transitions experienced by people who develop dementia whilst in the workforce. The aim is to assist individuals and their employers to better understand the needs of people living and working dementia as well as engage in appropriate actions that support choices and dignified transitions either within the context of employment or out of the workforce.

